# Altered methylation pattern of the *SRD5A2* gene in the cerebrospinal fluid of post-finasteride patients: a pilot study

**DOI:** 10.1530/EC-19-0199

**Published:** 2019-07-04

**Authors:** Roberto Cosimo Melcangi, Livio Casarini, Marco Marino, Daniele Santi, Samantha Sperduti, Silvia Giatti, Silvia Diviccaro, Maria Grimoldi, Donatella Caruso, Guido Cavaletti, Manuela Simoni

**Affiliations:** 1 Dipartimento di Scienze Farmacologiche e Biomolecolari, Università degli Studi di Milano, Milan, Italy; 2 Unit of Endocrinology, Department of Biomedical, Metabolic and Neural Sciences, University of Modena and Reggio Emilia, Modena, Italy; 3 Center for Genomic Research, University of Modena and Reggio Emilia, Modena, Italy; 4 Department of Medical Specialties, Azienda Ospedaliero-Universitaria di Modena, Modena, Italy; 5 Neurology Division, Papa Giovanni XXIII Hospital, Bergamo, Italy; 6 Experimental Neurology Unit and Milan Center for Neuroscience, School of Medicine and Surgery, University of Milano Bicocca, Monza, Italy

**Keywords:** 5 alpha-reductase, neuroactive steroids, finasteride, side effects, epigenetic changes

## Abstract

**Context:**

Post-finasteride syndrome (PFS) occurs in patients with androgenic alopecia after suspension of the finasteride treatment, leading to a large variety of persistent side effects. Despite the severity of the clinical picture, the mechanism underlying the PFS symptoms onset and persistence is still unclear.

**Objective:**

To study whether epigenetic modifications occur in PFS patients.

**Methods:**

Retrospective analysis of a multicentric, prospective, longitudinal, case–control clinical trial, enrolling 16 PFS patients, compared to 20 age-matched healthy men. Main outcomes were methylation pattern of *SRD5A1* and *SRD5A2* promoters and concentration of 11 neuroactive steroids, measured by liquid chromatography-tandem mass spectrometry, in blood and cerebrospinal fluid (CSF) samples.

**Results:**

SRD5A1 and SRD5A2 methylation analysis was performed in all blood samples (*n* = 16 PFS patients and *n* = 20 controls), in 16 CSF samples from PFS patients and in 13 CSF samples from controls. The *SRD5A2* promoter was more frequently methylated in CSF of PFS patients compared to controls (56.3 vs 7.7%). No promoter methylation was detected in blood samples in both groups. No methylation occurred in the *SRD5A1* promoter of both groups. Unmethylated controls compared to unmethylated *SRD5A2* patients showed higher pregnenolone, dihydrotestosterone and dihydroprogesterone, together with lower testosterone CSF levels. Andrological and neurological assessments did not differ between methylated and unmethylated subjects.

**Conclusions:**

For the first time, we demonstrate a tissue-specific methylation pattern of *SRD5A2* promoter in PFS patients. Although we cannot conclude whether this pattern is prenatally established or induced by finasteride treatment, it could represent an important mechanism of neuroactive steroid levels and behavioural disturbances previously described in PFS.

## Introduction

Finasteride is an inhibitor of 5 alpha-reductase (5α-R) type 1 and 2 enzymes, encoded by the *SRD5A1* and *SRD5A2* gene, respectively, with higher affinity for 5α-R type 2 in the human (1, 2). This enzyme converts testosterone into dihydrotestosterone (DHT) and progesterone into dihydroprogesterone (DHP) (3).

Clinically, finasteride is used to control the progression of benign prostatic hyperplasia and of androgenetic alopecia. Albeit being a well-tolerated and relatively safe drug, recent clinical studies showed sexual adverse effects (2, 4, 5, 6, 7). Interestingly, a small subset of patients using finasteride for androgenic alopecia, complains of persistent sexual side effects during and even after discontinuation of the treatment (2, 8, 9, 10, 11, 12, 13, 14, 15, 16, 17, 18, 19, 20). Beside adverse events in the sexual sphere, some patients interrupting the treatment by finasteride develop depression (16, 17, 18, 20, 21, 22, 23), reduction in self-confidence, decreased initiative and difficulty in concentration, forgetfulness or loss of short-term memory, irritability, suicidal thoughts, anxiety, panic attack, sleep problems, muscular stiffness and cramps, tremors, chronic fatigue, joint pain and muscular ache (18, 24, 25, 26). These various symptoms amount to the so-called post-finasteride syndrome (PFS). Two recent clinical studies objectivated impaired sexual function and major depression in PSF patients (17, 18). In addition, functional magnetic resonance imaging (MRI) showed abnormalities in the brain regions implicated in depression and regulating sexual arousal (17), and some evidence of neuropathy involving the peripheral neurogenic control of erection was produced (18).

PFS etiopathogenesis remains elusive. Three different clinical studies (18, 25, 26) demonstrated that finasteride treatment not only affects the steroids directly related with the enzyme 5α-R but has broad consequences on the levels of several important physiological regulators of the nervous function, such as neuroactive steroids, both in plasma and in cerebrospinal fluid (CSF). These results were confirmed in an animal model of PFS, showing that alterations in the levels of neuroactive steroids not only occurred in plasma and CSF but also in brain areas, such as cerebral cortex, cerebellum and hippocampus (27), associated to depressive-like behaviour, alterations in neurogenesis, gliosis, neuroinflammation and gut microbiota composition (28). A possible hypothesis for the persistent side effects may be epigenetic modifications occurring in PFS patients. Indeed, downregulation and hypermethylation of 5α-R were observed in the rodent nervous system and associated with inflammation and depression (29, 30), features that, as mentioned earlier, have been also observed in PFS model (28). In this setting, the evaluation of the methylation pattern remains challenging since it could change during adulthood and with ageing (31).

In this study, we hypothesised that inappropriate methylation of *SRD5A1* and/or *SRD5A2* gene promoters could be present in the central nervous system (CNS) of patients with PFS. No studies evaluated so far the methylation levels of these genes in a clinical setting. We compared *SRD5A1* and/or *SRD5A2* promoter methylation in DNA samples obtained from blood and/or CSF in PFS patients and controls and correlated the resulting epigenetic pattern with the neuroactive steroid levels previously found by us to be associated with major depression and sexual side effects (18).

## Materials and methods

### Study design and sample preparation

Sixteen patients affected by PFS, recruited through the ‘Italian network of finasteride side effects’, were included in this study. They were otherwise healthy men, aged 22–44 years, who reported persistent sexual and mental health side effects after the use of 1–1.25 mg of finasteride daily (Propecia, Proscar or generic finasteride) for androgenetic alopecia. Only subjects who had discontinued finasteride at least 3 months earlier were included. The study procedure was approved by the Ethics Committee of the San Gerardo Hospital (approval n.142/2012), Monza-Italy and the participating subjects provided their written informed consent before enrolment. This group of PFS patients was previously studied for psychiatric components, andrological assessment and neuroactive steroid levels in plasma and CSF (18).

PFS patients underwent both blood and CSF sampling for *SRDA51* and *SRDA52* gene promoter methylation analysis. The control group included 18 healthy individuals who underwent spinal anaesthesia for planned orthopaedic surgery of the lower limb at the San Gerardo Hospital of Monza. These subjects were otherwise healthy and never made used of finasteride. After written informed consent, CSF samples were collected. Two subjects of this group provided also a blood sample for DNA analysis. Moreover, the control group included additional 18 age-matched healthy donors, who provided only blood samples for DNA analysis. Control subjects never used finasteride. The mean age of healthy controls (40.8 ± 17.9 years) was not significantly different from PFS patients (34.5 ± 8.8 years) (*P* = 0.192).

### *SRD5A1* and *SRD5A2* promoter sequences

Sequences of* SRD5A1* and *SRD5A2* gene promoters served for designing primer probes to be used for methylation analysis. *SRD5A1* and *SRD5A2* promoter sequences were obtained by the online Eukaryotic Promoter Database (EPD; http://epd.vital-it.ch) and correspond to the database ID ‘SRD5A1_1’ and ‘SRD5A2_1’, respectively. The *SRD5A2* promoter sequence was previously described (32) and matched to that obtained by the EPD, confirming its reliability.

### DNA extraction and methylation analysis

Genomic DNA was isolated from blood and CSF samples by the automated extractor EZ1 Advanced XL (Qiagen) using the EZ1 DNA Blood and the EZ1 DNA Investigator Kits, respectively. We assumed that ependymal cells, of neuroectodermic origin and collected from CSF, are representative of the CNS, providing a source of genomic DNA without resorting to major clinical interventions. Due to the paucity of the cellular component in the CSF, DNA extraction was preliminarily validated, using a kit optimised for forensic purposes, to provide a yield of at least 2.8 ± 1.1 ng/µL from 4.3 ± 3.3 cells/µL (data obtained from four random, anonymised, sterile CSF validation samples, ranging from 100 to 500 µl of total volume).

DNA concentrations and purity were determined by the NanoDrop^™^ 2000 spectrophotometer (Thermo Fisher Scientific), while the methylation pattern was analysed using the Human Methylated & Non-methylated DNA Set (Zimo Research, Irvine, CA, USA). Serial dilutions of methylated DNA (0–100% range), provided by the supplier, were performed to generate the standard curve. Deamination of cytosines was obtained by bisulfite conversion of equal amounts of DNAs using the Methylamp DNA Modification Kit (EpiGentek, Farmingdale, NY, USA) and following the instructions provided by the Company. Quantitative methylation-specific PCR (Q-MSP) was performed for methylation analysis of CpG islands of *SRD5A1* and *SRD5A2* promoter regions. The methylation-specific primer pairs were designed by the MethPrimers Software (33) as follows: *SRD5A1* forward (Fwd): 5′-AGTTTTATATTTTTCGGGATTTTCG-3′ and reverse (Rev): 5′-CGCTTTAAACTTATTCCTAAACGAT-3′; SRD5A2 Fwd: 5′-AAGTTATGGAAGGATAGTTTAAGCG-3′ and Rev: 5′-TCTCAAAAATACAACCGCGAT-3′. The methylation-specific primers for the *ACTB* gene were previously validated (34) and used to provide the internal control. Reactions were performed using the Precision Melt Supermix (Bio-Rad Laboratories Inc.) following the supplier’s instructions and following settings: double-strand DNAs were pre-denatured 2 min at 95°C before 50 cycles of DNA denaturation at 95°C for 10 s, each followed by primer binding at 59°C for 30 s and extension at 72°C for 30 s. Samples and the standard curve were analysed in duplicate and the percentage of DNA methylation was calculated using the previously described 2^−ΔΔCt^ method (35) optimised for DNA methylation analyses (36, 37). Briefly, ΔCt values were calculated by subtracting the internal control Ct value to Ct of samples and standards. ΔΔCt was determined by subtracting the ΔCt of the 100% methylated DNA standard to the ΔCt of samples and standard dilutions. Standards were plotted on a X-Y graph, where the percentage of methylated DNA is at the X axis, while the 2^−ΔΔCt^ value is at the Y axis. These data were interpolated by linear regression and used for extrapolating the percentage of sample DNA methylation.

### Precision of the DNA methylation assay

Precision of the method was assessed by evaluating the coefficient of variation (%CV) (38) in relationship to the percentage of methylation. To this purpose, DNA with known percentages of methylation were created by serial dilution as reference standard and the coefficient of variation was calculated as the standard deviation divided by the mean (×100).

Cut-off for defining the methylated status was fixed at 10% and values below this threshold were considered as unmethylated DNA, as previously proposed (39). %CV of each sample was extrapolated from the reference standard curve and used for evaluating whether it was reliably determined as falling above the methylation cut-off value. All samples are reliably classified as ‘methylated’ or ‘unmethylated’, according to the cut-off value fixed at 10% CV.

### Statistical analysis

Results are expressed as mean ± s.d. Methylation status of the *SRD5A1* and *SRD5A2* gene promoters were expressed as percentage of methylated DNA extrapolated from the standard curve.

Kolmogorov–Smirnov test was used to evaluate continuous neuroactive steroid data distribution. Neuroactive steroid levels in plasma and CSF previously obtained (18) were compared between *SRD5A2* unmethylated controls, methylated and unmethylated PFS patients using Kruskall–Wallis test followed by *post hoc* Dunnet test. The correlation between neuroactive steroid levels and percentage of promoter gene methylation was evaluated using Spearman’s Rho test.

The degree of erectile dysfunction and the depressive status of PSF patients was previously reported (18) and was reassessed here by comparing *SRD5A2* unmethylated and methylated PFS patients using Pearson’s chi-squared test. Similarly, the total score of Beck Depression and Anxiety Inventories (BDI, BAI) and K-10 was compared between patients with and without *SRD5A2* methylation, using Mann–Whitney’s *U* test.

Statistics was performed using ‘Statistical Package for the Social Sciences’ software for Macintosh (version 20.0; SPSS Inc.).

## Results

DNA methylation analysis of *SRD5A1* and *SRD5A2* was successfully performed in all blood samples (*n* = 16 PFS patients and *n* = 20 controls), in 16 CSF samples from PFS patients and in 13 CSF samples from controls.

The *SRD5A1* gene promoter was completely unmethylated in all samples analysed, irrespective whether blood- or CSF-derived DNA.

Similarly, the* SRD5A2* gene promoter resulted unmethylated (i.e. methylation level <10%) in all blood-derived DNA samples, both in controls and in PFS patients. In CSF-derived DNA, the* SRD5A2* gene promoter resulted methylated in 9 out of 16 PFS patients and in 1 out of 13 controls (*P* = 0.006, Pearson’s chi-square test, Table 1). Interestingly, the control subject with positive CSF *SRD5A2* methylation was a man affected by normotensive hydrocephalus. In the PFS patients with positive *SRD5A2* methylation in CSF, the methylation levels ranged from 15.4 to 100% (mean: 40.3%, median: 31.8%), while the unique control sample resulted to be positive to the test showed 58.0% methylation.

**Table 1:** *SRD5A2* promoter methylation in DNA extracted from cerebrospinal fluid of PFS patients and controls.

	Controls (*n*)	PFS patients (*n*)	Total (*n*)
Unmethylated* SRD5A2*	12 (92.3%)	7 (43.7%)	19
Methylated* SRD5A2*	1 (7.7%)	9 (56.3%)	10
Total (*n*)	13	16	

Data were evaluated by Pearson’s chi-square test,* P* < 0.01.

Neuroactive steroid levels in these subjects were reported previously (18). In order to assess whether *SRD5A2* methylation affects neuroactive steroid levels, here we compared CSF concentrations in unmethylated controls vs unmethylated and methylated PFS patients (Table 2). Within the limits of the low subject number and of the sensitivity of the LC-MS/MS method for some analytes, the results showed some statistically significant differences (Table 2) indicating higher pregnenolone in unmethylated *SRD5A2* controls vs unmethylated *SRD5A2* PFS patients. On the contrary, higher testosterone levels were reported in unmethylated *SRD5A2* PFS patients vs unmethylated *SRD5A2* controls. The 5α-reduced metabolites of testosterone and progesterone were also affected. In particular, higher DHT levels were observed in unmethylated *SRD5A2* controls vs unmethylated *SRD5A2* PFS patients (Table 2). In addition, DHP levels were significantly higher in unmethylated *SRD5A2* controls not only vs unmethylated *SRD5A2* PSF patients but also vs methylated *SRD5A2* PSF patients (Table 2).

**Table 2:** Neuroactive steroid CSF levels in patients and controls according to the *SRD5A2* methylation status.

	Unmethylated *SRD5A2* controls (*n* = 12)	Unmethylated* SRD5A2* PFS patients (*n* = 7)	Methylated *SRD5A2* PFS patients (*n* = 9)	*P* value
Pregnenolone	0.39 ± 0.25	0.09 ± 0.05^a^	0.31 ± 0.46	**0.008**
Progesterone	0.11 ± 0.14	0.05 ± 0.01	0.34 ± 0.82	0.111
Dihydroprogesterone	3.07 ± 1.54	0.25 ± 0.00^a,b^	1.00 ± 1.28^a^	**0.001**
Isopregnanolone	0.11 ± 0.03	0.10 ± 0.00^#^	0.32 ± 0.63	0.531
Tetrahydroprogesterone	0.11 ± 0.03	0.25 ± 0.29	0.10 ± 0.00^b^	0.275
Dehydroepiandrosterone	0.29 ± 0.08	0.33 ± 0.05	0.51 ± 0.58	0.217
Testosterone	0.23 ± 0.85	2.53 ± 2.35^a^	1.26 ± 1.48	**0.028**
Dihydrotestosterone	0.15 ± 0.12	0.05 ± 0.01^a^	0.49 ± 1.13	**0.029**
3α-Diol	0.05 ± 0.00^#^	0.07 ± 0.04	0.64 ± 0.86	0.081
3β-Diol	0.05 ± 0.00^#^	0.05 ± 0.00^b^	0.25 ± 0.57	0.305
17β-Estradiol	0.4 ± 0.06	0.02 ± 0.00^b^	0.22 ± 0.56	0.676

Neuroactive steroid levels were previously reported (18). Detection limit was 0.1 pg/µL for isopregnanolone and tetrahydroprogesterone, 0.05 pg/µL for progesterone, and 3β-diol, 0.02 pg/µL for 17β-estradiol. Data are expressed as pg/µL (mean ± s.d.). Data were analysed by Kruskall–Wallis test followed by *post hoc* Dunnet test (^a^ vs unmethylated controls, *P* < 0.05). Bold indicates statistical significance.

^b^Denotes analytes for which the assay detection limit was used for the statistical analysis.

In PFS patients, neuroactive steroid CSF levels were not significantly related to the percentage of* SRD5A2* gene promoter methylation (*P* > 0.05, Spearman’s Rho test).

We next evaluated whether the clinical parameters reported earlier (18) were related to the* SRD5A2* methylation status. No differences were observed in the degree of erectile dysfunction (*P* = 0.362, Pearson’s chi-square test). Accordingly, the five international indexes of erectile function (IIEF-15) domains, identified as erectile function, orgasm, sexual desire, intercourse satisfaction and overall satisfaction, did not differ between groups (*P* = 0.710, *P* = 0.456, *P* = 0.535, *P* = 0.805 and *P* = 0.620, respectively). Similarly, the depressive status previously demonstrated in these PFS patients (18) did not change between *SRD5A2* methylated and unmethylated subjects, considering K-10 (*P* = 0.890) and BDI and BAI (*P* = 0.475 and *P* = 0.485, respectively). The depression and anxiety degree frequency did not change between two groups (*P* = 0.270 and *P* = 0.176, respectively).

## Discussion

In this study, we demonstrate that *SRD5A2* promoter methylation in PFS patients is different between tissues of neuroectodermic and mesodermic origin. Although DNA extraction was insufficient in five CSF samples, a significant difference between PFS patients and controls in *SRD5A2* promoter methylation of nervous system (i.e., in CSF) was demonstrated, while no differences were seen for the *SRD5A1* gene, which resulted unmethylated in all samples analysed. Interestingly, in PFS patients, *SRD5A2* methylation was detected in CSF DNA samples in 56% of the cases, while it was absent in blood DNA, demonstrating a tissue-specific epigenetic gene silencing. The only CSF sample showing *SRD5A2* methylation in controls was detected in one man with normotensive hydrocephalus. Methylation in CSF but not in blood may be not surprising, since methylation is a tissue- and cell-specific process (40, 41, 42). The low number of cases studied here does not allow definitive conclusions, but we can speculate that PFS primarily affects nervous system via methylation of *SRD5A2*, which, in turn, may be related to the clinical picture of PFS patients (i.e., major depression and evidence of neuropathy involving the peripheral neurogenic control of erection) (18, 43).

In this context, it is important to note that in human prostate, methylation of *SRD5A2* is regulated by DNA methyltransferase 1 (DNMT1) (44) and that, as demonstrated in animal models, forebrain deletion of DNMT1 (45) or its pharmacological inhibition (44, 46) had antidepressant-like properties.

The reason for the persistence of PFS symptoms even long after drug withdrawal in PFS patients remains a mystery. The molecular mechanism of 5α-R inactivation by finasteride is known since two decades (47), likely resulting in persistent enzymatic inhibition over time (47, 48), but this does not fully justify the long-lasting alteration of the neuroactive steroid levels, unless several months/years are required to completely wash-out the drug from the CSF. Assuming that the 5α-R protein blocked by finasteride cannot become functional again, in this study, we hypothesised that new *SRD5A1* and *SRD5A2* transcripts should be produced and new 5α-R protein should be available once the drug is interrupted, provided the gene promoters are not methylated and silenced. This should be evident from the CSF steroid concentrations in relation to the methylation pattern. Significant changes in neuroactive steroid levels in this group of PFS patients were published earlier, demonstrating a derangement of these levels upstream testosterone production (18). In fact, PFS patients showed higher serum and CSF testosterone levels than healthy controls, while other neuroactive steroids, also including DHT, were suppressed (18). In the present study, the comparison of CSF steroid levels subdividing the patients according to their *SRD5A2* methylation status provided some further insights. In unmethylated *SRD5A2* PFS patients, we can assume that type 2 5α-R is regularly expressed in CNS and finasteride can act on it. Indeed, in these patients the blockade of 5α-R activity by finasteride results in significant accumulation of testosterone and significant reduction of 5α-reduced metabolites DHT and DHP, the latter being even undetectable (Table 2). On the other hand, in methylated *SRD5A2* patients, finasteride might act, even if with lower affinity, predominantly or only on the type 1 5α-R, since *SRD5A1* should be expressed. This is suggested by the only partial reduction of DHP, while testosterone and DHT levels were not significantly different from controls. The significant decrease in pregnenolone in *SRD5A2* unmethylated PFS patients compared to controls indicate that the blockade of type 2 5α-R activity impacts the very early steps of neurosteroidogenesis as well, although with unknown mechanism. These results, however, must be interpreted with extreme caution, given the low number of subjects analysed. In animal models, it was demonstrated that neurosteroidogenesis may proceed in the brain converting progesterone in DHP and further into tetrahydroprogesterone (THP; allopregnanolone) (3) via a pathway similar to the so-called backdoor pathway of 5α-reduction, using type 1 5α-R (49). Therefore, the changes in neuroactive steroid levels in CSF of PFS patients demonstrated earlier (18), might be due to inhibition of type 2 5α-R only in the subjects with unmethylated *SRD5A2*, while in those with methylated* SRD5A2*, type 1 5α-R could be the target of finasteride. Whether the sexual and psychiatric symptoms documented in these patients are related to methylation of *SRD5A2* cannot be concluded by this study, as no significant differences could be demonstrated in dependence thereof, probably due to the low number of subjects.

Presently, it is impossible to establish whether the *SRD5A2* methylation pattern in PFS patients is set during early embryo development or results from finasteride treatment itself. DNA methylation is imprinted during gamete production and reprogrammed before embryo implantation (50). Besides the role in determining tissue-specific gene expression, epigenetic mechanisms regulate the individual-specific differentiation of pluripotent stem cells into neurons, suggesting that methylation impacts the pathogenesis of psychiatric disorders (51). Recent findings in rat models confirmed that altered epigenetic processes, consisting in abnormal DNA methyltransferase protein levels and increased global DNA methylation levels, are linked to anxiety- and depression-like behaviour (52). Our finding of high levels of methylated *SRD5A2* in CNS cells of a normotensive hydrocephalus individual, displaying an epigenetic status at odds with all other control subjects, further supports the link between DNA methylation pattern and disorders of the nervous system (53). If abnormal *SRD5A2* promoter methylation is established prenatally, these subjects could be predisposed to develop the PFS and alteration of neuroactive steroid levels in the brain. In this case, treatment by finasteride might precipitate a dormant depressive phenotype linked to such specific epigenetic pattern, resulting in the clinical picture described previously (18, 43). Before finasteride treatment, the impairment of 5α-R type 2 in the nervous system would be partly compensated by the activity of the 5α-R type 1, found always unmethylated and thereby expressed. On the other hand, the native DNA methylation pattern may change during adulthood and it is susceptible to ageing (54, 55), diseases (56, 57, 58, 59), environmental factors and inflammation (60, 61). Most importantly, similar effects might be triggered by prolonged exposure to certain drugs, such as amphetamine and methamphetamine (62), inducing behavioural abnormalities. We cannot exclude that some *SRD5A2* promoter methylation is determined by finasteride (or other drugs) exposure in adulthood in some subjects developing PFS, although the mechanism of such predisposition remains obscure. Given that epigenetic programming impacts the neuroendocrine response during the adult life (63), we could hypothesise that finasteride impairs DNA methylation targeted to the nervous system, leading to altered neurosteroid CSF levels and leading to major depression and neuropathy described in PFS patients (18, 43), but this remains highly speculative.

In conclusion, our results demonstrate methylation of *SRD5A2* promoter in a tissue-specific manner in PFS patients. Whether this epigenetic pattern is established prenatally or induced by finasteride treatment cannot be concluded but this study pinpoints the relevance of this specific methylation pattern and its correlation with levels of neuroactive steroids and their effects (18, 43). Animal models might be useful to elucidate the link between the use of finasteride, epigenetic changes, neuroactive steroid levels and behavioural disturbances described previously in PFS patients (27, 28).

## Declaration of interest

The authors declare that there is no conflict of interest that could be perceived as prejudicing the impartiality of the research reported.

## Funding

The authors thank the Post-Finasteride Foundation for financial support.
